# A dose-finding, long-term study on the use of calcium chloride in saline solution as a method of nonsurgical sterilization in dogs: evaluation of the most effective concentration with the lowest risk

**DOI:** 10.1186/s13028-014-0063-1

**Published:** 2014-10-14

**Authors:** Raffaella Leoci, Giulio Aiudi, Fabio Silvestre, Elaine A Lissner, Fabio Marino, Giovanni M Lacalandra

**Affiliations:** Department of Emergency and Organ Transplantation (DETO), Section of Veterinary Clinic and Animal Production, University of Bari Aldo Moro, SP per Casamassima km 3, 70010 Valenzano, BA Italy; Parsemus Foundation, PO Box 2246, 94702 Berkeley, CA USA; Department of Veterinary Science, University of Messina, Polo Universitario dell’Annunziata, Viale Annunziata, 98168, Messina, Italy

**Keywords:** Calcium chloride, Canine, Dog, Nonsurgical sterilization, Testicular injection, Population management

## Abstract

**Background:**

Canine overpopulation is a global issue with serious health and welfare implications. Nonsurgical methods of sterilization could yield positive impacts on this problem, but no long-term data on such methods are available. The objective of the current investigation was to determine the effects of intratesticular injections of calcium chloride dihydrate (CaCl_2_) in saline in dogs over a one year period. Five concentrations (0%, 10%, 20%, 30%, 60%) of CaCl_2_ in saline were administered via intratesticular injection to groups of 10 dogs each. Total sperm count and motility, blood levels of testosterone, and side effects were examined at 0, 2, 6, and 12 months post-injection (PI). Testicular size and semen volume were examined at 0 and 12 months PI.

**Results:**

Total sperm count, semen volume and testosterone showed significant dose-dependent decreases upon treatment with 10%-60% CaCl_2_ compared with either the control group (0% CaCl_2_) or baseline for each treatment group. Azoospermia was achieved for at least 12 months PI in 60% and 80% of treated dogs after administration of a 10% and 20% CaCl_2_, respectively. Treatment with 30% or 60% CaCl_2_ resulted in azoospermia in 100% of dogs, but more side effects were observed, while no side effects were noticed at lower doses. For each treatment group, testosterone levels had decreased an average of 35%-70% at 6 months following treatment. However, testosterone levels rebounded by the 12-month time point in all groups except the highest dosage group (60% CaCl_2_), which remained at the low end of physiological range throughout the study. Sperm motility dropped to zero or near zero in all dogs treated with CaCl_2_. Testicular size was significantly smaller at 12 months PI for all groups when compared to baseline.

**Conclusions:**

This first long-term study confirms reports of the efficacy of CaCl_2_ sterilization. However, at dosages free of adverse events, calcium chloride in saline may not provide permanent sterilization as previously believed. Future work should explore optimized solvents to increase the permanence of the well-tolerated 20% formulation.

## Background

Canine overpopulation is an intractable problem globally despite local efforts to control population growth. In many developing countries, free-roaming dogs cause serious health and welfare problems with resulting socio-economic, ecological, political, and ethical issues. Therefore, the development of effective fertility control measures is a high priority. Many communities that aspire to mass surgical sterilization find it prohibitively resource-intensive. As a result, lifelong housing in shelters or mass euthanasia campaigns continue to be used to control dog populations, but the effects prove to be minor, not cost-effective, and often against international animal welfare regulations [1,2]. Thus, several alternatives to surgical sterilization have been explored. For contraception of stray male dogs, desirable methods need to be permanently effective, minimally invasive, simple, rapid, and inexpensive, with potential for adoption in large-scale animal birth control programs [3].

Intratesticular injection of chemicals is a non-surgical method for contraception of male dogs [4]. This approach has been investigated for more than five decades [5]. An ideal chemical sterilizing agent for stray dogs would be one that effectively arrests spermatogenesis, androgenesis, and libido, while lacking toxicity and serious side effects [6]. Many compounds have been studied (e.g., glycerol, zinc compounds, and chlorhexidine) [6-10]. While many potential candidates possess some anti-spermatogenic or anti-fertilizing activity, most compounds do not eliminate gonadal sources of testosterone [4]. In contrast, calcium chloride dihydrate (CaCl_2_), a salt used for several medical purposes [11], represents a promising chemical sterilant [12,13]. CaCl_2_ can be dissolved in water [12,13], in alcohol [12,13], or lidocaine solution [2,14,15]. Several investigators [2,14] have reported that a single, bilateral, intratesticular injection of CaCl_2_ solution resulted in induction of permanent (i.e., irreversible) chemosterilization, including cessation of sperm production and decreased testosterone in male dogs. These earlier studies evaluated the effectiveness of CaCl_2_ on testicular histology within two months of injection, and the concentration of sterilant used per testicle was frequently correlated with the animal body weight [2,12-14]. However, use of a concentration per body weight approach is not practical for application in the field, as stray or wild animals would have to be weighed and customized concentrations of sterilant prepared according to the weight of individual dogs. In order to determine an effective, permanent nonsurgical sterilization method for dogs, long-term data are needed evaluating and comparing the reproductive parameters and safety of various concentrations and formulations.

In the current study, the effects of a range of concentrations of CaCl_2_ on reproductive physiology and canine health were measured in male dogs over a 12-month period. The dosage of CaCl_2_ per testicle was based on testicular width. The goal was to compare the impact of different CaCl_2_ concentrations in physiological saline on sperm production, blood levels of testosterone, testicular size, adverse events, and long-term fertility.

## Methods

### Animals

Fifty mature, healthy, mixed-breed, owned male dogs 2 to 5 years of age (mean = 3.6 years, SD = 1.1 years), weighing 18 to 24 kg (mean = 21.1 kg, SD = 2.2 kg) were used. This study was conducted at a private shelter with cooperation of the shelter owner. Dogs remained at the shelter throughout the study. Good health status was confirmed by routine blood tests and clinical examinations. To assess the fertility of the dogs, an andrological examination including physical and ultrasonographic examinations and evaluation of semen quality was performed before the start of the study. All dogs showed sexual interest in a bitch in estrus.

Dogs received routine deworming and vaccinations. The dogs were not subjected to changes in habits during the study, were fed standard commercial dog food twice daily, and were given water *ad libitum*. Dogs were housed in groups of three in a comfortable primary enclosure with outdoor runs. Indoor space had temperature maintained above 15°C and below 26°C and relative humidity ranging from 30% to 70%.

Investigations were conducted in accordance with the Principles for the Care and Use of Research Animals promulgated by the European Union. The Italian Ministry of Health (Progetto di Ricerca corrente 2009 IZS SI 11/09: “Randagismo applicazione e valutazione di metodi innovativi per il controllo delle nascite”) approved this study.

### Experimental protocol

Dogs were randomly divided into five groups (A-E) of 10 dogs per group. At day 0 (T_0_), semen evaluation and blood testosterone analysis were performed. Appropriate concentrations of CaCl_2_ sterilant were prepared as described below. After light sedation with an intramuscular (IM) injection of 5-10 mg of acepromazine maleate (Prequillan, Fatro, Italy) per 10 kg, testicular width was measured with a caliper. Based on the scrotal width, the dosage of the experimental or control solution was injected in each testicle. Groups of dogs received 10% (group A), 20% (group B), 30% (group C), or 60% (group D) of CaCl_2_ or only saline solution (control group E). At 2, 6 and 12 months (T_2_, T_6_, T_12_ respectively) post-injection (PI), semen was evaluated for total sperm count and progressive motility and blood testosterone concentrations were determined. Semen was evaluated for volume and testicles for width at T_0_ and T_12_. Throughout the trial, the dogs were under regular clinical observation.

### Preparation and intratesticular injection of CaCl_2_ solution

Four solutions of CaCl_2_ were prepared. For each experimental group, 10 g (Group A), 20 g (Group B), 30 g (Group C), or 60 g (Group D) of CaCl_2_ powder (Sigma Aldrich Corporation) was dissolved in a physiological saline solution (Sodio Cloruro Bieffe Medital 0.9%, Grosotto [SO], Italy) to a final volume of 100 ml, mixed, and sterilized in Falcon tubes. Sterile physiological saline served as the control solution. Prior to injection, fur from long-haired dogs was trimmed from the scrotum after the dogs were sedated. The scrotal skin was then gently washed with saline solution and disinfected with a sodium hypochlorite solution (0.05% Amukine, Angelini, Italy).

Animals received single, bilateral intratesticular injections at a dose corresponding to the testicular width, according to a prior research protocol (personal communication, Dr. Kuladip Jana). Animals with scrotal diameters of 19-22 mm received 0.8 ml injections, whereas animals with scrotal diameters of at least 23 mm wide received 1 ml injections. The 10 animals in the control group E, which all had testicular widths of at least 23 mm, received single, bilateral intratesticular injections of 1 ml of sterile saline solution.

To inject the solution, a 22 gauge/30 mm needle was inserted from the caudal pole of each testis approximately 5 mm from the epididymal tail, and directed towards the opposite pole of the testis. The solution was carefully deposited along the entire route from the proximal to the distal end by linear infiltration [16]. The injection was completed at a moderately slow pace (5-12 sec depending on volume) and all the solution was injected before the needle was withdrawn from the testis to prevent seepage of the solution from the injection site. When the injection was 1/3 complete, the pressure applied to the testicle was reduced to accommodate the volume of fluid being injected. After the injection the needle was removed quickly while simultaneously releasing the testicle. No further intratesticular injections were performed on any of the animals throughout the rest of the study.

### Total sperm count volume and motility

Semen was collected by digital manipulation of the penis [17] using plastic cones (artificial vaginas) (IMV Technologies, Italia). Semen was immediately collected in three fractions using sterile graduated tubes. To evaluate the effect of the chemical sterilization on semen characteristics, semen volume was recorded and analyzed. The second sperm-rich fraction of the ejaculate was examined within 30-60 min by computer-assisted sperm analysis (CASA) (IVOS Version 12.2; Hamilton Thorne Biosciences Inc., Beverly, MA, USA). This interval had been validated in preliminary studies not to significantly influence semen parameters (data not shown). During this period, the semen was stored at 37°C. A 4 μl aliquot was mounted on a 4-chamber Leja® slide (Microptic S.L.) at 37°C and evaluated for sperm concentration and percentage of progressive motile sperm [18,19]. Total sperm count was then obtained.

### Assay for serum testosterone

Between 8:00 a.m. and 8:30 a.m. at T_0_ to T_12_ dogs received subcutaneous injections of 1,000 international units of human chorionic gonadotrophin (hCG) (Creative Biomart, CD Inc.) in order to stimulate gonadal testosterone levels [20-22]. Blood was collected from the saphenous vein of each dog 120 min after the hCG injections. A portion of blood was allowed to stand for 10-15 min at 4°C, then centrifuged at 1,500 g for 10 min at 4°C prior to aspiration of serum. Serum samples were stored at -20°C until thawed and assayed for testosterone concentrations by a chemiluminescence technique (Immulite Immunoassay System, Siemens).

### Clinical examination

All animals were subjected to routine clinical examination during the T_0_ to T_12_ period. After the chemical sterilization procedure, the animals were observed continuously for the first 72 hours, followed by daily observation for up to 15 days, with clinical examination again at 2, 6, and 12 months. Clinical examination included recording body weight, blood counts, general attitude, appetite, rectal temperature, scrotal and inguinal integument, palpation of testes, and heart and respiratory rates.

### Measurement of testicular width

Testicular width of each testicle was measured as an index of testicular size [23]. Caliper measurements were obtained on each testicle at T_0_ and at T_12_.

### Adverse effects management

In case of severe testicular side effects, dogs were surgically castrated. The surgically excised testes were sampled for histological examination. The testicles were fixed in Bouin’s fixative and routinely processed for paraffin embedding by a previous de-scaling treatment (CAL-EX Decalcifier, Fisher Chemical, USA), followed by cutting on a microtome. Five-micron sections were stained with hematoxylin-eosin and von Kossa stain.

Representative microscopic fields were selected and digitized using software for image analysis. Morphometric measures were performed on scanned images to identify and quantify the extent of the lesions, by estimating changes in ratios of different cell types (including germ cells and stroma) and between intracellular components (nucleus/cytoplasm, volume, and morphology).

### Statistical analysis

Clinical data, treatment and time were included in the statistical analyses and average and standard deviation (SD) (mean ± SD) calculated when appropriate. Statistical analyses were conducted using *Statistica* (StatSoft, Inc. Tulsa, OK, USA).

Repeated measures analysis of variance (ANOVA), with Time as the within factor and Group as the between factor, were used to evaluate three measurements in the five groups (A-E) across the four timepoints (T_0_, T_2_, T_6_, T_12_) for semen characteristics and testosterone or two timepoints (T_0_, T_12_) for semen volume and testicular width. If the overall test had statistical significance, then planned comparisons were conducted. Dunnett’s test for comparison to control group E was used, as well as univariate or multivariate planned comparisons to determine if the measures changed after treatment and if the treated groups differed from the control group E. A two-tailed significance level of *P* < 0.05 was identified.

Eight dogs underwent surgical castration and as a result had missing data for testosterone levels, semen volume, sperm motility, and testicular measurements (see Results).

## Results

### Clinical observation

All animals in the study tolerated the insertion of the needle and intratesticular injection well. Only a small number of dogs showed signs of slight discomfort during the CaCl_2_ or saline injection: 2% (1/50) of injected dogs vocalized, and 4% (2/50) had abdominal muscle contraction at needle puncture of the scrotum. Even though injection was performed carefully, seepage occurred in 2% of the dogs (1/50), and the seepage was wiped away immediately with dry gauze. No adverse effects were noticed during the 12 months study in the dog where seepage occurred.

During the first two weeks after the CaCl_2_ injection, dogs in groups A and B did not exhibit any agitation, fever, or marked inflammatory swelling of the testis. However, all dogs (experimental and control groups) had a slight increase in the firmness of the testes on palpation from 24 hours PI and until PI days 3-7. Atrophy of the testes gradually progressed in groups A and B dogs from 1 week to approximately 1.5 months PI leaving a small fibrotic remnant.

In groups C and D, some dogs showed sign of discomfort and licked the site of injection. Within two weeks, 2 dogs (20%) in group C and 6 dogs (60%) in group D developed a testicular fistula. These 8 dogs underwent therapy with anti-inflammatory drugs and antibiotics and surgical castration. These dogs were excluded from the study.

No variation in body weight was recorded in any of the groups. Significant variation in the blood parameters was not found except for dogs excluded from the study. Dogs developing testicular fistula had leukocytosis indicating an inflammatory reaction (i.e., 21,000 white blood cells/ml[mean]).

### Adverse reactions

**Figure 1:**
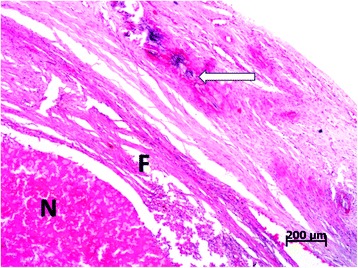
**Photomicrograph of testicular tissue from a dog in group C performed at day 15 post-injection.** Testicular tissue from an orchiectomized dog in group C with abundant necrosis (N) surrounded by a fibrous connective tissue reaction (F) and calcification (arrow) on the periphery of the testicle. Tissues were stained with hematoxylin and eosin using standard methods.

**Figure 2:**
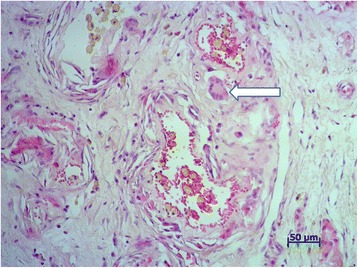
**Photomicrograph of testicular tissue from an orchiectomized dog in group C.** The arrow shows interstitial infiltration of giant cells. Tissues were stained with hematoxylin and eosin using standard methods.

Two dogs of group C and six of group D showed an inflamed scrotum by 72 h PI which developed into a scrotal ulcer and fistula in the following days. Surgical castration and scrotal ablation was conducted on day 15 PI. Histological examination of testicular tissue from orchiectomized dogs in group C showed massive necrosis surrounded by fibrous connective tissue and peripheral calcification (Figure 1). Sparse calcium deposits were present at the periphery of the testicles while the remaining tissue showed severe tubular degeneration. At higher magnification, the seminiferous epithelium was constituted of one or two cell layers lining the tubular lumen. Moreover, plasma cells, macrophage infiltration and the presence of numerous multinucleated giant and epithelioid cells were found inside the seminiferous tubules (Figure 2).

**Figure 3:**
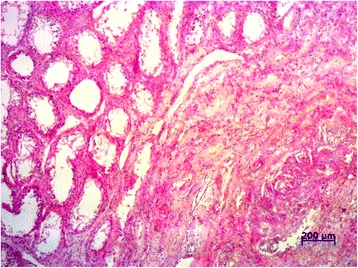
**Photomicrograph of testicular section from dogs in group D.** Testicular tissue from orchiectomized dogs in group D showing severe tubular degeneration with seminiferous tubules appearing as empty spaces (on the left) and diffuse interstitial fibrosis. Tissues were stained with hematoxylin and eosin using standard methods.

Histological examination of testicular tissue from orchiectomized dogs in group D showed large necrotic areas often surrounded by a thick granulomatous/fibrous reaction. The remaining parenchyma was constituted of seminiferous tubules severely degenerated and surrounded by diffuse interstitial fibrosis. Abundant interstitial calcium deposits, hemorrhages and rare giant cells, often inside the tubular lumen, were also present (Figure 3).

### Semen volume, total sperm count and progressive sperm motility

Evaluation of the semen volume collected at T_0_ and T_12_ revealed a significant interaction among groups (Group × Time interaction: F = 9.84, *P* < 0.001). Semen volume declined significantly over time for experimental groups A-D (*P* < 0.001) but not for the control group E (*P* = 0.322). There was no significant difference between groups at T_0_, but semen volume for all treatment groups was lower than group E at T_12_ (F = 22.7, *P* < 0.001). Changes in semen volume for each group also varied in a dose dependent manner, with the maximum decrease in group D of 37.6% (group A T_0_: 3.5 ± 0.44, T_12_: 3.2 ± 0.59; group B T_0_: 2.6 ± 0.36, T_12_: 2.0 ± 0.44; group C T_0_: 3.4 ± 0.48, T_12_: 2.4 ± 0.49; group D T_0_: 3.1 ± 0.65, T_12_: 1.9 ± 0.36; group E T_0_: 3.1 ± 0.56, T_12_: 3.3 ± 0.56).

Statistical analyses revealed that the total sperm count differed significantly across time points and between groups (Group × Time interaction: F = 76, *P* < 0.001) due to significant declines in the experimental groups (but not control group) after treatment with CaCl_2_. No significant difference between groups was found at T_0_: the mean total sperm count (×10^6^) was 332.9 ± 51.2 in group A, 342.4 ± 35.0 in group B, 336.3 ± 30.1 in group C, 328.0 ± 32.5 in group D, and 335.9 ± 34.9 in the control group E. The total sperm count in the control group E also remained stable at T_2_, T_6_ and T_12_ (340 ± 23.3, 313.5 ± 40.6, 311.4 ± 21.4, respectively). However, after treatment with CaCl_2_, all experimental groups had significantly lower total sperm counts than the control group E (F = 5879, *P* < 0.001) and compared to their T_0_ value (*P* < 0.001) (Table 1).

**Table 1:** **Effects on reproductive parameters 1 year after intratesticular injection of calcium chloride or saline**

**Intratesticular injection**	**Total sperm count (×10** ^**6**^ **)**	**Sperm motility (%)**	**Serum testosterone concentration (ng/dL) after hCG stimulation**	**Testicular width (mm)**
Saline Control (E)	311.4 ± 21.4	80 ± 5.8%	735.2 ± 186.4	24.9 ± 2.1
10% CaCl_2_ (A)	29.8 ± 38.7^**+**^	10 ± 5.2%^**+**^	487.7 ± 144.2	12.9 ± 1.4
20% CaCl_2_ (B)	7.5 ± 16.2^**++**^	5 ± 2.1%^**++**^	461.0 ± 286.6	12.3 ± 0.9
30% CaCl_2_ (C)	0	–	299.3 ± 170.7	12.4 ± 1.5
60% CaCl_2_ (D)	0	–	125.7 ± 48.9	12.0 ± 0.7

Effects on reproductive parameters in male dogs were measured 1 year after a single, bilateral intratesticular injection of calcium chloride. Some of the male dogs in groups C and D also underwent surgical castration. Data for dogs in groups A, B, and E and dogs in group C and D that did not undergo surgical castration are expressed as mean ± SD. +60% of the dogs were azoospermic, and 40% were oligospermic. ++80% of the dogs were azoospermic, and 20% showed severe oligospermia.

– Undetectable.

Total sperm count and progressive motility were inversely associated with the dosage of CaCl_2_ administered. All experimental dogs were azoospermic at T_2_ and T_6_. At T_12_, 6 (60%) dogs in group A remained azoospermic, and 4 dogs (40%) were severely oligospermic (74.5 ± 6.6, range 65-80) (Table 1). The mean total sperm count (×10^6^) in group A was 29.8 ± 38.7 at T_12_. In group B, 8 (80%) of dogs remained azoospermic, and 2 (20%) of dogs were severely oligospermic (37.5 ± 10.6, range 30-45) at T_12_ (Table 1). All dogs in group C and group D remained completely azoospermic at T_12_.

As expected, progressive sperm motility reflected the results of total sperm counts. At T_0_, motility was 90 ± 4.7% in group A, 75 ± 7.1% in group B, 80 ± 8.5% in group C, 70 ± 0.0% in group D, and 80 ± 5.8% in the control group E. Motility of sperm in the control group E averaged 80 ± 5.8% at all times tested. It was only possible to measure sperm motility in the treatment groups at T_12_ for dogs in groups A (10% motility in all 4 dogs) and B (5% motility in both dogs). Statistical analysis of sperm motility was not possible due to the lack of variability among the groups.

Semen collection was easily performed at all time points and no differences were noticed in relation to the CaCl_2_ treatment groups. Semen collection was not performed in dogs that underwent surgical castration.

### Assay of serum testosterone

The mean hCG-stimulated testosterone levels at T_0_ were within physiological range (100-1,000 ng/dl) [24] for all dogs: 591.0 ± 140.1 in group A, 581.8 ± 223.2 in group B, 654.8 ± 233.1 in group C, 413.0 ± 138.1 in group D, and 721.2 ± 176.2 in the control group (E). A single intratesticular injection of CaCl_2_ decreased serum testosterone concentrations in a dose-dependent manner (Table 2). A significant interaction between Group and Time was found (F = 7.40, *P* < 0.001). The control group’s testosterone level did not change significantly over time, but each experimentally treated group had significantly decreased testosterone levels (*P* < 0.006) (Tables 1 and 2). Compared to pre-injection concentration (T_0_), testosterone levels decreased on average 35.4% in Group A, 41.7% in Group B, 70.1% in Group C, and 60.1% in Group D. Treatment with 30% or 60% CaCl_2_ showed higher efficacy in decreasing testosterone levels than did treatment with 10% or 20% CaCl_2_. The testosterone levels of dogs treated with 60% CaCl_2_ remained near the lower limit of the physiological range throughout the study, whereas testosterone in groups treated with lower concentrations of CaCl_2_ had increased at T_12_.

**Table 2:** **Mean (+/- SD) testosterone levels (ng/dL) in serum following hCG stimulation**

**Group**	**T**_**0**_	**T**_**2**_	**T**_**6**_	**T**_**12**_	**Ave T**_**2**_ **-T**_**12**_
10% CaCl_2_ (A)	591.0 ± 140.1	468.8 ± 105.4	189.5 ± 79.0	487.7 ± 144.2	382.0 ± 167.0^a,b^
20% CaCl_2_ (B)	581.8 ± 223.2	257.0 ± 179.3	300.0 ± 211.3	461.0 ± 286.6	339 ± 107.5^a,b^
30% CaCl_2_ (C)	654.8 ± 233.1	128.6 ± 50.5	159.9 ± 66.8	299.3 ± 170.7	195 ± 90.8^a,b^
60% CaCl_2_ (D)	413.1 ± 138.1	209.8 ± 25.7	103.5 ± 13.5	125.7 ± 48.9	146 ± 56.0^a,b^
Saline Control (E)	721.2 ± 176.2	663.6 ± 154.3	640.4 ± 149.9	735.2 ± 186.4	679.7 ± 49.4

T_0_, T_2_, T_6_, and T_12_ indicate the time-points (time zero, 2 months, 6 months, and 12 months, respectively) following intratesticular injection. Data are expressed as mean ± SD. After injection of calcium chloride, levels of testosterone in serum decreased: a = significant within group difference comparing T_0_ to T_2_-T_12_; b = significant between group difference comparing group A-D to control group E at T_2_-T_12_.

### Measurement of testicular width

At T_12,_ the scrotal widths had decreased significantly by approximately 50% in all of the CaCl_2_-treated groups compared with the control group E. The mean values of testicular width (mm) at T_0_*vs* T_12_ were 24.6 ± 2.5 *vs* 12.9 ± 1.4 in group A; 23.8 ± 1.1 *vs* 12.3 ± 0.9 in group B; 24.3 ± 1.2 *vs* 12.4 ± 1.5 in group C; 24.3 ± 1.2 *vs* 12.0 ± 0.7 in group D; and 24.9 ± 2.1 *vs* 24.9 ± 2.1 in the control group (E) (Table 1). As with sperm count and testosterone, analysis of testicular width showed an interaction between Group and Time (F = 243, *P* < 0.001). Testicular width in experimental groups differed from the E group (*P* < 0.001) due to reduced testicular width measurements at T_12_ for the experimental groups (F = 525.7, *P* < 0.001). As expected, the testicular widths in the control group E remained unchanged over time.

## Discussion

The results of this long-term study have important implications for nonsurgical sterilization of male dogs. Treatment with CaCl_2_ resulted in atrophy of the testicles and significantly lower semen volume, total sperm count, testosterone levels and testicular measurements compared with the control group. Although a complete fertility evaluation of characteristics such as sperm morphology was not performed, the complete absence of sperm seen at higher doses indicates likely contraceptive effect.

A dose-dependent effect on the reproductive characteristics was found with increasing concentrations of CaCl_2_ resulting in more durable azoospermia, but simultaneously leading to higher risk of complications.

Measuring testicular size and injecting a predetermined concentration of CaCl_2_ is expected to be more practical than weighing individual dogs and preparing customized injection solutions as reported in previous studies [2,12-14]. Our results indicate that our approach is useful and effective and could be applied to a field neutering campaign or management of a population of stray dogs.

The minor discomfort during injection of CaCl_2_ or saline noticed in a few dogs was caused by the needle and intra-testicular pressure. Afferent nerve endings associated with pain sensation are located only on the scrotal skin and in the capsule of the testis rather than within the testicular parenchyma. Therefore, these nerve endings may have been stimulated as intra-testicular pressure increased during and immediately following injection [24]. The injections were well tolerated, an important welfare consideration.

We found that intratesticular injection of CaCl_2_ in a saline solution as a nonsurgical sterilizing agent had a dose-dependent effect on dogs. Intratesticular injection of CaCl_2_ achieved and maintained infertility for at least 6 months. However, 60% of the dogs receiving 10% CaCl_2_ and 20% of the dogs receiving 20% CaCl_2_ had regained some testicular activity by 12 months PI. Although the maximum response in terms of contraception was seen with injections of 30% CaCl_2_ and 60% CaCl_2_, these high doses resulted in a higher risk of complications that might necessitate emergency surgery (which was performed in 20% and 60% of the dogs receiving these doses, respectively). Use of such high concentrations is contraindicated, as complications cannot be managed in stray dogs or during high-volume neutering campaigns.

In agreement with the findings of previous studies, a 20% solution of CaCl_2_ was the most effective dose that resulted in elimination of sperm in most dogs but did not result in serious complications [2,13,15]. Previous short-term investigations reported complete sterilization with various dosages and formulations of CaCl_2_ [2,12-15]. Importantly, our study showed a certain level of sperm production one year after treatment with the lower concentrations (10% and 20%). Although the partial recovery of sperm production may not be sufficient for restoration of fertility in male dogs, we cannot exclude the possibility that recovery of fertility could occur. Levels of serum testosterone also began to increase at T_12_ in groups A-C. These results point to the importance of long-term studies to gauge the effectiveness of chemical castration methods, which is critical to their use in stray dog population management programs.

## Conclusions

Our study confirmed the effectiveness of an intratesticular CaCl_2_ injection as a promising sterilization agent. A practical dosing system by testicular width was demonstrated and a 20% solution of CaCl_2_ was identified as the most effective dose without risk of serious complications. However, in contrast to previous reports that assumed permanent sterilization based on testicular histology, in conducting the first long-term study we found that some testicular function had returned after one year at all but the highest dose. Given the considerable promise of this method, further studies should explore use of different solvents (alcohol, lidocaine) with the goal of boosting CaCl_2_ efficacy at the well-tolerated 20% dose.
